# Enhancing the Low Oral Bioavailability of Sulpiride via Fast Orally Disintegrating Tablets: Formulation, Optimization and In Vivo Characterization

**DOI:** 10.3390/ph13120446

**Published:** 2020-12-05

**Authors:** Hesham M. Tawfeek, Yasser A. Hassan, Mohammed F. Aldawsari, Mohamed H. Fayed

**Affiliations:** 1 Department of Industrial Pharmacy, Faculty of Pharmacy, Assiut University, Assiut 71526, Egypt; 2 Department of Pharmaceutics, Faculty of Pharmacy, Delta University for Science and Technology, Gamasa 35523, Egypt; yasserhassan4543@yahoo.com; 3 Department of Pharmaceutics, College of Pharmacy, Al-Bayan University, Baghdad 10001, Iraq; 4 Department of Pharmaceutics, College of Pharmacy, Prince Sattam Bin Abdulaziz University, Al-kharj 11942, Saudi Arabia; moh.aldawsari@psau.edu.sa (M.F.A.); m.fayed@psau.edu.sa (M.H.F.); 5 Department of Pharmaceutics and Industrial Pharmacy, Faculty of Pharmacy, Fayoum University, Fayoum 63514, Egypt

**Keywords:** sulpiride, fast disintegrating tablets, dopamine antagonist, factorial design, Prosolve^®^, bioavailability

## Abstract

Sulpiride (SUL) is a dopamine D_2_-receptor antagonist used for management of GIT disturbance and it has anti-psychotic activities based on the administered dose. SUL undergoes P-glycoprotein efflux, which lead to poor bioavailability and erratic absorption. Therefore, the objective of this research was an attempt to enhance the oral bioavailability of SUL via formulation of fast disintegrating tablets (SUL-FDTs) with a rapid onset of action. A 3^2^ full-factorial design was performed for optimization of SUL-FDTs using desirability function. The concentration of superdisintegrant (X_1_) and Prosolv^®^ (X_2_) were selected as independent formulation variables for the preparation and optimization of SUL-FDTs using direct compression technique. The prepared SUL-FDTs were investigated regarding their mechanical strength, disintegration time, drug release and in vivo pharmacokinetic analysis in rabbits. The optimized formulation has hardness of 4.58 ± 0.52 KP, friability of 0.73 ± 0.158%, disintegration time of 37.5 ± 1.87 s and drug release of 100.51 ± 1.34% after 30 min. In addition, the optimized SUL-FDTs showed a significant (*p* < 0.01) increase in C_max_ and AUC(_0–∞_) and a relative bioavailability of about 9.3 fold compared to the commercial product. It could be concluded that SUL-FDTs are a promising formulation for enhancing the oral bioavailability of SUL concomitant with a fast action.

## 1. Introduction

Sulpiride, SUL, belongs to a group of drugs that selectively inhibit the dopaminergic receptors [1,2]. It has numerous uses as an anti-depressant, anti-psychotic, anti-ulcers and in the treatment of gastrointestinal tract (GIT) disturbances, e.g., vomiting and nausea, depending on the administered dose [1]. The physicochemical characteristics of the drug reveal that it has a low aqueous solubility and limited permeability hence it is classified through the biopharmaceutical classification system under the category IV [3]. Hence, a reported low oral bioavailability has been reported previously [4]. Furthermore, it was also proved that SUL was a substrate for P-gp, P-glycoprotein, a protein located in different sections of GIT which plays a role in drug efflux further lowering its bioavailability [5]. In addition, SUL has an absorption window in the upper GIT [6]. All of these factors lead to its observed poor oral bioavailability and erratic oral absorption.

Different approaches have been investigated to improve the bioavailability of drugs substrates to P-gp via co-administration of P-gp inhibitors. However, most of these inhibitors have issues regarding their safety and possible drug interactions, especially the first and second generation inhibitors [7]. Furthermore, the third generation P-gp inhibitors like elacridar and tariquidar show a higher activity at low concentration. However, their cost is still an issue for effective product formulation and commercialization. All of this finally obliged scientists to look for more appropriate means to solve such types of problem. Previous work in our laboratory sought to enhance drug oral bioavailability via formation of a gastric-retained microsponge in an attempt to delay drug release to overcome the absorption window. This study succeeded in enhancing the drug oral bioavailability compared to the commercial capsule product [8], however, some aspects regarding gastro-retention of the formulation related to patients stomach’s physiological state and feeding conditions are still issues requiring more efficient formulations applicable to wide ranges of patients. Ibrahim et al., have produced solid-lipid nanoparticles of SUL with an ultrasonic melt-emulsification method. Their study revealed higher intestinal permeability with anticipated higher bioavailability [9], however, very little investigation has been performed to tackle the problem of the P-gp efflux pump.

Fast dissolving tablets (FDTs) have shown promise for solving many problems associated with oral drug absorption. They offer fast action, rapid onset, high absorption while also showing precise dosing and ease of manufacture [10]. Rapid tablet disintegration and dissolution in the oral cavity enables drugs to be absorbed in the oral cavity, pharynx and esophagus, thus avoiding the gastric absorption. In addition, they are a good alternative for drugs that show a first pass effect [11].

The aim of this work was to formulate SUL in FDTs for a rapid onset effect, which is preferred for patients suffering from nausea and vomiting as well as elderly patients. In addition, it represents a possible alternative to avoid the P-gp efflux in the intestine with a possible enhancement of SUL oral bioavailability. Fast disintegrating tablets were prepared via the direct compression technique. Different excipients used were investigated for their compatibility performance with the drug using differential scanning calorimetry. In addition, a 3^2^ full-factorial design was performed to optimize the SUL-FDTs formulation utilizing the Design-Expert software. The prepared tablets were characterized for their weight variation, content uniformity, mechanical strength, in vitro disintegration, and in vitro drug release. Moreover, pharmacokinetic parameters were investigated and compared with the marketed SUL product (Dogmatil^®^ capsule, Sanofi Aventis, Egypt) in an animal model (New Zealand rabbits).

## 2. Results and Discussion

### 2.1. Compatibility Study

**Figure 1:**
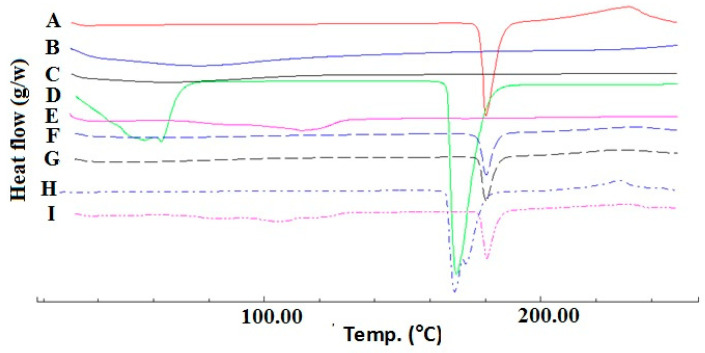
DSC thermograms of SUL (**A**); Prosolv^®^ (**B**); croscarmellose (**C**); mannitol (**D**); magnesium stearate (**E**) and their respective physical mixture ((**F**–**I**), traces) at 1:1 *w*/*w* ratio.

DSC thermograms of SUL, croscarmellose, Prosolv^®^, mannitol and magnesium stearate are presented in Figure 1. It was clear that SUL has a sharp pure endothermic peak at 179.94 °C (Figure 1A) which is related to the melting of crystalline SUL. Croscarmellose alone and Prosolv^®^ alone showed broad peaks ranging from 50–100 °C that are related to the release of adsorbed water molecules [10]. Mg stearate showed also a characteristic melting curve at approximately 115.0 °C, which indicates its melting [12]. In addition, mannitol alone has a sharp melting endotherm at 170.0 °C. Physical mixtures of SUL with the investigated excipients showed the characteristic melting endotherm of SUL at approximately the same melting temperature of pure SUL, concomitant with a reduction in intensity which was attributed to the dilution effect resulting from the mixing with excipient [10,13]. In case of mannitol, both characteristic melting peaks were observed with a slight down shift of SUL melting peak to 174.5 °C. The slight shift of the SUL melting endotherm could be attributed to the partial solubility of SUL in the melted mannitol as it has a lower melting point or due to the dilution effect and geometry of the mixing process during sample preparation. Mora et al., showed some sort of interaction between dehydroepiandrosterone and mannitol resulted in the appearance of new peaks and an increase in the enthalpy of fusion of the physical mixture peak (which were not detected in our results). The authors stated that such an interaction is not necessarily an incompatibility [14].

Similarly, Ghosh et al., showed also a shift in temperature of a rizatriptan benzoate physical mixture with mannitol (1:1 wt ratio) from 167.5 °C to 175.2 °C and also concluded the absence of any incompatibility [15]. From the abovementioned results, it could be confirmed the absence of any physical or chemical interaction between SUL and the selected excipients. So, they could be further processed for tablet manufacturing.

### 2.2. Effect of Independent Variables on SUL-FDTs Properties

#### 2.2.1. Weight Uniformity and Content Uniformity

The weight uniformity and SUL contents in the prepared SUL-FDTs are presented in Table 1. It was found that the average tablet weight ranged from 195.6 ± 3.2 to 201.8 ± 1.6 mg. All the prepared tablets showed acceptable weights according the USP standards for the uniformity of tablet weight.

Nevertheless, a small observed variation in tablet weight could be attributed to variations in the bulk density of the compressed powder [16]. These results suggested the proper flowing properties of the prepared formulations. Besides, the drug content ranged from 97.66 ± 1.45% to 100.30 ± 1.74% with RSD < 6%. Additionally, the acceptance value (AV) of content uniformity was less than 15.0. This result revealed that all prepared SUL-FDTs met the USP criteria for dosage form uniformity [17].

#### 2.2.2. Hardness and Friability

As shown in Table 1 the SUL-FDTs hardness ranged from 3.25 ± 0.25 to 4.90 ± 0.19 KP. Regression analysis (Table 2) showed that tablet hardness was significantly influenced by both the superdisintegrant concentration (*p* < 0.0197) and Prosolv^®^ concentration (*p* = 0.0005) with a dominant effect of Prosolv^®^ concentration as evident by its high sum of squares (0.0228 for superdisintegrant and 0.3083 for Prosolv^®^). In addition, the tablet hardness was found to be positively correlated with the Prosolv^®^ concentration and negatively with the superdisintegrant concentration according to the sign of their coefficient estimates (+0.2267 for Prosolv^®^ and −0.0167 for superdisintegrant). 

**Figure 2:**
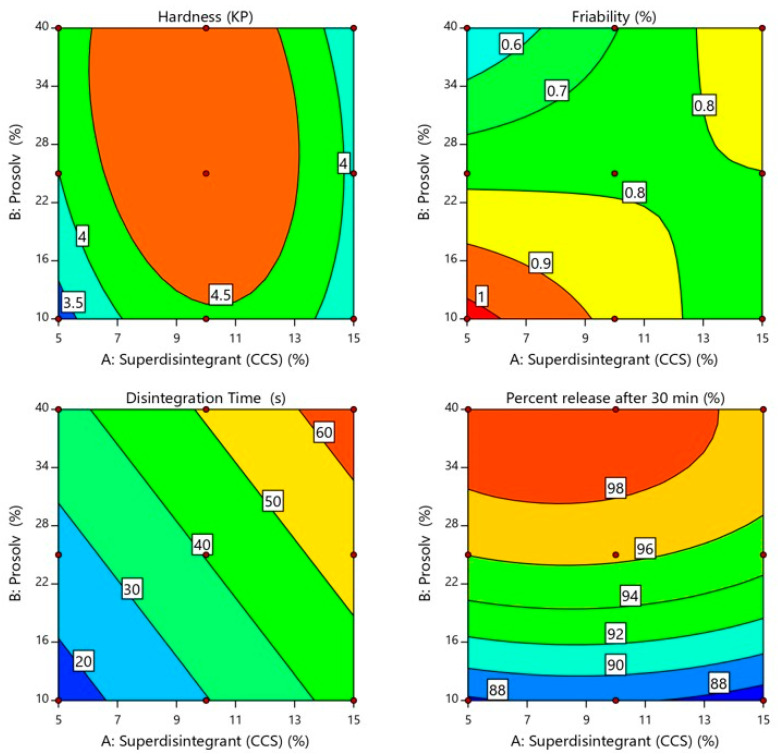
Influence of independent variables on hardness, friability, in vitro disintegration time and percent drug release after 30 min. of SUL-FDTs.

Furthermore, the tablet hardness was increased as Prosolv^®^ concentration increased due to the binding activity of Prosolv^®^ as depicted in Figure 2 [18]. On the other hand, the tablet hardness was positively correlated with the superdisintegrant concentration between 5% and 10% and the tablet hardness was increased from 3.25 to 4.9 KP. However, by increasing the superdisintegrant concentration over 10%, the tablet hardness decreased significantly from 4.9 to 3.56 KP due to quadratic effect of superdisintegrant [19]. Therefore, higher tablet hardness appeared at a combination of high level of Prosolv^®^ and medium level of superdisintegrant as shown in the lower middle of the contour plot (Figure 2). On the other hand, the two-way interaction between X_1_ and X_2_ also had a significant (*p* = 0.0008) effect on tablet hardness in a negative direction according to the negative sign of coefficient estimate (−0.2325).

The regression analysis demonstrated that the quadratic model was valid for tablet hardness. The quadratic equation that explained the influence of X_1_ and X_2_ on tablet hardness is as the follows (Equation (1)):

Hardness (KP) = 4.93 − 0.0167 × X_1_ + 0.2267 × X_2_ − 0.2325 × X_1_ X_2_ − 1.0 × X_1_^2^ − 0.2867 × X_2_^2^ (1)

Friability is another important tablet attribute related to tablet mechanical strength [16]. The idea behind friability test is to determine the ability of the tablet to withstand abrasion during production and handling [20]. As shown in Table 1 the SUL-FDTs friability ranged from 0.378 ± 0.157 to 1.01 ± 0.438%. All the prepared SUL-FDTs come in accordance with the USP limitation for friability test, being less than 1.0%. The friability was found to be positively correlated with the superdisintegrant concentration and negatively with Prosolv^®^ concentration according to the positive and negative sign of their coefficient estimates (+0.0137 for superdisintegrant and −0.090 for Prosolv^®^). However, these two variables had no significant effect on SUL-FTDs friability as shown by the results of regression analysis (*p* = 0.8859 for superdisintegrant and *p* = 0.3657 for Prosolv^®^) as pointed in Table 2. In addition, the two-way interaction between X_1_ and X_2_ had no significant (*p* = 0.1727) effect on tablet friability. Figure 2 reveals that SUL-FDTs friability increased as superdisintegrant concentration increased, while, the concentration of Prosolv^®^ showed an opposite effect on SUL-FDTs friability. Therefore, it is obvious that the lowest friability appeared at a higher level of Prosolv^®^ and lower level of superdisintegrant as observed from the contour plot in the higher left side corner (Figure 2).

The regression analysis demonstrated that the two-factor interaction (2FI) model was valid for tablet friability. The regression equation that explained the influence of X_1_ and X_2_ on tablet friability has the following expression (Equation (2)):

Friability (%) = 0.7849 + 0.0137 × X_1_ − 0.090 × X_2_ + 0.1762 X_1_ X_2_ (2)

#### 2.2.3. In Vitro SUL-FDTs Disintegration

Disintegration of tablets is the rate-determining step for drug release, especially for poorly water-soluble drugs. It is initiated by penetration of fluid in the pores of a compressed tablet followed by mechanical fragmentation into smaller fragments [21]. This leads to a significantly higher dissolution rate due to the increased surface area available for this process [22]. It was observed that all the prepared formulations had a rapid disintegration time that ranged from 15.16 ± 0.89 to 66.33 ± 1.55 s as shown in Table 1. According to the FDA (2008), orodispersible tablets should disintegrate within 30 s using the USP disintegration test [23]. Formulations F1 and F2 showed a disintegration time of less than 30 s. The fastest disintegration time of 15.16 s was seen for F1 containing the lowest level of superdisintegrant and Prosolv^®^ (5.0 and 10.0%, respectively) as displayed in the lower left corner of the contour plot (Figure 2). Moreover, the regression analysis showed that the concentration of superdisintegrant and Prosolv^®^ had a significant positive effect (*p* < 0.012 and *p* < 0.0356, respectively) on SUL-FDTs disintegration time, according to the sign of coefficient estimate (+14.17 and +10.75, respectively) as shown in Table 2. On the other hand, the mutual interaction between X_1_ and X_2_ had no longer any effect on tablet disintegration time (linear model). As provided in Figure 2 the disintegration time of prepared SUL-FDTs was directly proportional to the concentration of both superdisintegrant and Prosolv^®^ with a rapid and sharp increase in the disintegration time as the level of superdisintegrant and Prosolv^®^ increased from 5.0% to 15.0% and from 10.0% to 40.0%, respectively. However, the concentration of superdisintegrant was considered the most influential variables on SUL-FDTs disintegration time, as evident by their sum of square values (1204.45 for superdisintegrant and 693.59 for Prosolv^®^) and coefficient of estimate values (14.17 for superdisintegrant and 10.75 for Prosolv^®^). It was reported that croscarmellose sodium accelerates tablet disintegration due to its ability to absorb a high amount of water when exposed to an aquatic environment [20]. Swelling, wicking and strain recovery are also reported as possible mechanisms of disintegration using croscarmellose sodium [24]. Therefore, the combined effect of water absorption and swelling create high disintegration pressure resulted in shattering of tablets and subsequently rapid disintegration. On the other hand, increasing the concentration of Prosolve^®^ resulted in a remarkable extend of tablet disintegration time. This could be attributed to the presence of colloidal silicon dioxide, as one of constituents of Prosolve^®^, which reduced penetration of water into the tablets as well as reduction of water available for the deployment of the microcrystalline cellulose activity as disintegrant [25].

The regression analysis demonstrated that the linear model was valid for SUL-FDTs disintegration time. In addition, the regression equation that explained the influence of X_1_ and X_2_ on SUL-FDTs disintegration time is as the following equation (Equation (3)):

Disintegration time (s) = 40.35 + 14.17 × X_1_ + 10.75 × X_2_ (3)

#### 2.2.4. In Vitro Release Study

**Figure 3:**
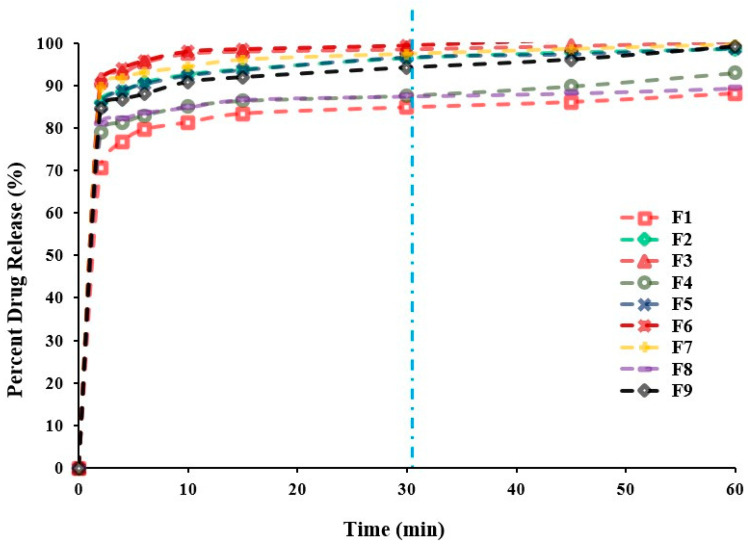
In vitro SUL release profiles from SUL-FDTs based on 3^2^ Full Factorial Design. Because of overlapping, error bars are omitted for clarity.

Dissolution profiles are one of critical quality attributes of tablets which affect a drug’s bioavailability and hence, its pharmacological action [22]. Figure 3 represents the in vitro release profiles for all different formulations at pH 6.8. Generally, the results of the in vitro release study demonstrated that all tablets exhibited a considerably higher SUL dissolution rate due to the increased surface area available for dissolution after rapid disintegration of the tablets into smaller granules and fine particles [21]. Besides, all formulations showed acceptable release profiles with respect to the USP standards for immediate release tablet, since they released more than 80.0% of SUL within 30 min (Table 1). It can be observed that the concentration of Prosolv^®^ had a significant (*p* < 0.0004) impact on the SUL percentage released from FDTs after 30 min, whereas, a minor effect (*p* < 0.1383) of superdisintegrant concentration on SUL release from FDTs was observed upon regression analysis investigation, as depicted in Table 2. Further, the mutual interaction between X_1_ and X_2_ had no significant (*p* = 0.6205) effect on drug release. Moreover, coefficient estimate values demonstrated that the changes in the level of Prosolv^®^ had a remarkable impact on SUL release in a positive direction according to the positive sign of coefficient of estimate (+5.40). The microcrystalline cellulose content of Prosolv^®^ can swell on contact with water. This swelling cause the tablet to disintegrate with subsequent increase in SUL release due to the higher surface area available of the formed small fragments [21,26]. Conversely, the level of superdisintegrant exerted a negative effect on dissolution of SUL, this effect was indicated by the negative sign of parameter estimate (−0.618). Therefore, an increase in the level of superdisintegrant (more than 10%) decreased the SUL release due to gelling effect and subsequent increase in viscosity [26]. Figure 2 represents the effects of Prosolv^®^ and superdisintegrant on SUL release. It was found that, the percentage of SUL released after 30 min was strongly affected by the concentration of Prosolv^®^ with a sharp increase in SUL release from 87.89 ± 2.05% to 99.39 ± 0.96% as the level of superdisintegrant increased from 5.0% to 10.0% (Table 1). Meanwhile, when the level of superdisintegrant was more 10.0%, the SUL release decreased from 99.39 ± 0.96% to 94.15 ± 1.16% (Table 1). These results indicated that the release of SUL from FDTs was significantly influenced by the formulation variables.

The regression analysis data demonstrated that the quadratic model was valid for drug in vitro release profile. The regression equation that explained the influence of X_1_ and X_2_ on drug dissolution is as the following equation (Equation (4)):

Percentage SUL release = 96.31 − 0.618 × X_1_ + 5.40 × X_2_ − 2.07 X_1_ X_2_ − 0.901 × X_1_^2^ − 2.7 × X_2_^2^ (4)

#### 2.2.5. Determination of Optimized Formulation Using Desirability Function

**Figure 4:**
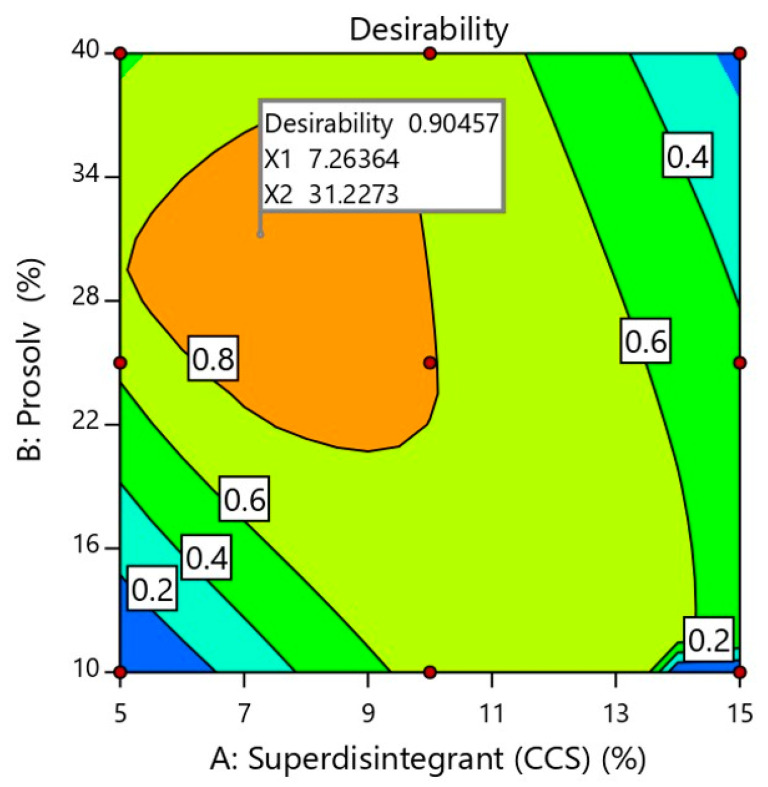
Optimization plot showing the influence of independent formulation variables on overall desirability. X1 and X2 represents the concentration of superdisintegrant and Prosolv^®^ in the optimized SUL-FDTs formulation.

In the optimization step, the software integrates all dependent responses in such a way that the optimized formulation has the right balance of all the investigated and desired attributes [11]. For a successful orally disintegrating tablet formulation, high mechanical strength, rapid disintegration and acceptable drug release are required. Therefore, numerical optimization using a desirability approach was done to predict the optimized formulation. In the present work, tablet mechanical strength and SUL release were maximized while tablet disintegration time was minimized. The selected constrained values for the optimized formulation were given in Table 3. Based on the contour plot of desirability (Figure 4), it was expected that the most desirable mechanical strength, disintegration time and SUL release would be obtained with superdisintegrant and Prosolv^®^ quantities of 7.26% *w*/*w* and 31.22% *w*/*w*, respectively. The predicted optimized formulation containing aforesaid quantities of superdisintegrant and Prosolv^®^ was prepared and physically characterized as previously demonstrated. The prediction error of the model was determined by comparing experimental (actual) and predicted values. As displayed in Table 4 the obtained experimental values of the optimized formulation were in close agreement with the predicted values. The values of relative error were found to be less than the 5.0% that confirmed the validity and predictability of the applied design [27].

#### 2.2.6. In Vivo Pharmacokinetic Analysis of Optimized Formulation

**Figure 5:**
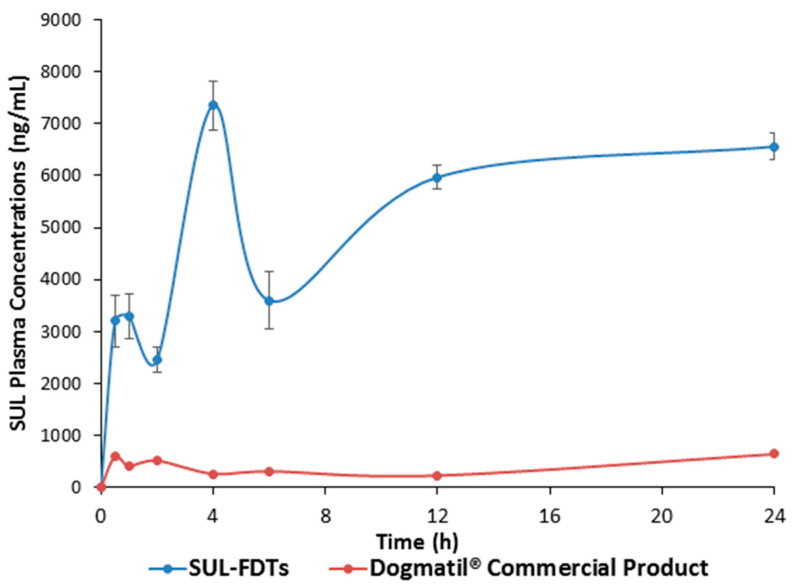
Plasma concentration—time profile of SUL for both SUL-FDT and Dogmatil^®^ commercial product after administration of optimized SUL-FDT formulation and Dogmatil^®^ to male Newzealand rabbits (*n* = 5, each value is the average ± S.D.).

The different pharmacokinetic parameters for both optimized SUL-FDTs and commercial product as well as the plasma concentration time curve are presented in Table 5 and Figure 5, respectively. 

The pharmacokinetic parameters showed multiple plasma peak concentrations as previously reported for SUL absorption [28,29,30]. This phenomenon revealed that SUL has different sites located in GIT for its absorption. Furthermore, this behavior is also observed with other related benzamide derivatives. Actually, after rapid disintegration and dissolution, part of the drug could be absorbed from the oral cavity, which is responsible for the first absorption phase that appeared in the first 30 min. Further, the remaining amounts were transferred through the GIT and delivered to the absorption site in the upper part of small intestine which probably responsible for the further absorption phases. The optimized SUL-FDTs showed significantly higher (*p* < 0.01) C_max_ and AUC compared with the commercial SUL product. According to the obtained values of AUC from time zero to infinity for both SUL-FDTs and Dogmatil^®^ (Table 5), it was found that the relative bioavailability showed a 9.3-fold enhancement compared with the commercial product, which interestingly means that the oral bioavailability of SUL increased more than 9-fold. Hence, altering the drug route of administration from oral capsules to FDTs plays a significant role in enhancement of SUL bioavailability.

It is also worth mentioning that the initial SUL plasma concentration after 0.5 h and 1.0 h was found to be 3205 ± 498.8 ng/mL and 3299.66 ± 432.02 ng/mL, respectively which is significantly higher compared to the SUL concentrations obtained from the commercial product at the same time points. This high concentration is suitable for rapid controlling GIT disorders such as nausea and vomiting and hence, an advantage of FDTs formulation. In addition, the significantly (*p* < 0.01) higher C_max2,3_ delineates a high SUL plasma concentration for a long period of time which was also proved from long MRT value of approximately 15.0 h. Finally, it was interesting to note the significant enhancement in SUL oral bioavailability in comparison to commercial oral formulation Dogmatil^®^ via the FDTs dosage form as well as an anticipated rapid onset of action. In addition, the FDTs could be a promising alternative formulation for drug substrates for P-gp efflux to enhance their absorption and hence, their bioavailability.

## 3. Materials and Methods

### 3.1. Materials

Sulpiride was kindly donated from Memphis for Pharmaceutical and Chemical Industry (Cairo, Egypt). Croscarmellose sodium (VIVASOL^®^) and silicified microcrystalline cellulose (PROSOLV^®^ SMCC) was obtained from JRS Pharma (Rosenberg, Germany). Mannitol was obtained from Roquette America (Keokuk, Iowa, USA). Magnesium stearate was obtained from by El-Nasr Pharmaceutical Chemicals Co. (Cairo, Egypt). Experimental rabbits with an average body weight of 1.5 ± 0.2 kg (adult males, New Zealand type) were supplied by the College of Medicine animal house of Assiut University (Assiut, Egypt). Anti-coagulant, Cal-Heparine^®^ injection ampoules, containing heparin were delivered by Egyptian Amoun Company (Al Obour, Egypt). All other chemicals used were of analytical grade and used as received.

### 3.2. Compatibility Study

Compatibility investigation between SUL, croscarmellsoe sodium, Prosolv^®^, mannitol and magnesium stearate was investigated via differential scanning calorimetry (DSC) as mentioned before [31,32]. Briefly, SUL and the investigated excipients were thoroughly mixed (1:1 *w*/*w* wt. ratio). Five mg of each sample were placed into an aluminium pan with a capacity of fifty µL and thickness of 0.1 mm. Further, pans were press-sealed using aluminium cover of about 0.1 mm in thickness and then, samples were scanned from 25 °C to 300 °C in a DSC-50 equipment (Shimadzu, Kyoto, Japan). Scanning was performed under nitrogen flow of 40 mL/min and heating rate of 10 °C min^−1^. TA50 software (Shimadzu) was utilized to analyse the scanned thermograms.

### 3.3. Experimental Design and Statistical Analysis

According to preliminary studies a 3^2^ full-factorial design using the Design-Expert 11 software (Stat-Ease, Inc. Minneapolis, MN, USA) was carried out to explore the influence of superdisintegrant, croscarmellose sodium, (X_1_) and two in one highly processed excipient, Prosolv^®^, (X_2_) as independent formulation variables on the critical attributes of SUL-FDTs prepared by direct compression method. As displayed in Table 6 each variable was investigated at three levels namely low (−1), medium (0) and high (+1). Table 7 shows the full matrix of the design generated by software.

Formulation at the center point was carried out in triplicate at different days to validate the design and prevent the experimental error. Using Design-Expert software, ANOVA test was done to assess the influence of independent variables on the studied dependent responses at 95% level of significance. The general polynomial equation applied for 3^2^ factorial design is as follows (Equation (5)):

Response variable (Y) = β_0_ + β_1_ X_1_ + β_2_ X_2_ + β_3_ X_1_ X_2_ + β_4_ X_1_^2^ + β_5_ X_2_^2^ (5)

where, β_0_ is the arithmetic mean response of all runs β1, β2, β3, β4 and β5 are regression coefficient of estimate of the independent variables X_1_ and X_2_. X_1_ X_2_ and X_1_^2^ & X_2_^2^ represent the interaction and quadratic effect, respectively. The relative error was determined according to the following equation (Equation (6)):

(6) $$\mathrm{Relative}\;\mathrm{error}\;\left(\%\right)=\left(\;\frac{\left|\mathrm{Predicted}\;\mathrm{value}-\mathrm{Experiment}\;\mathrm{value}\right|}{\mathrm{Predicted}\;\mathrm{value}}\;\right)\times 100$$

### 3.4. Preparation of SUL-FDTs

SUL-FDTs were formulated through the direct compression method. The composition of the prepared tablets was presented in Table 8. Croscarmellose sodium (CCS) was used as a superdisintegrant [10,33]. Additionally, Prosolv^®^ is a multifunctional excipient composed of microcrystalline cellulose and colloidal silicone dioxide. It has a binder-filler, glidant and lubricant activities with improved blending and high intrinsic flowability [34]. Moreover, mannitol was used as a filler and a sweeting agent [20]. Briefly, 50 mg of SUL and the calculated amounts of each other excipients, according the experimental design, were mixed in a glass bottle and the drug content in the prepared tablets was used to verify the efficiency of mixing. Each formulation was finally mixed with 1.0% *w*/*w* magnesium stearate and compressed using Erweka single punch tablet machine (Germany) with 8.0 mm die set and tablet weight of 200.0 mg. Machine compression speed was approximately 50–60 tablet/ minute. The produced SUL-FDTs were collected and stored in tightly closed polyethylene container for further studies.

### 3.5. Characterization of SUL-FDTs

#### 3.5.1. Weight Uniformity

The weight uniformity of the formulated SUL-FDTs was performed according to the USP 38 [17]. Twenty tablets were randomly selected from each batch, then the average tablet weight and respective standard deviation (SD) values were calculated.

#### 3.5.2. Content Uniformity

SUL content was determined in the prepared FDTs according to the USP 38 guidelines [17]. The AV, acceptance value, was calculated according to the following equation (Equation (7)):

(7) AV=(X−M)+KS

where, *X* is the mean value of drug content, *S* is the standard deviation and *K* is a constant value either equal 2.4 for 10 dosage units. Briefly, 10 tablets were chosen randomly, each tablet was powdered and dissolved in methanol, solutions were filtered using a membrane filter (0.45 μm, Agilent Technologies, Santa Clara, CA, USA). Finally, SUL content was determined spectrophometrically at 290 nm as the wave length of maximum absorbance (model 6305, JENWAY, Staffordshire, UK). Then, the AV value was calculated according to the provided equation.

#### 3.5.3. Tablet Hardness

SUL-FDTs hardness was measured using Erweka hardness tester (Erweka; Frankfurt, Germany). Six tablets were used in this test and the average value ± SD was recorded.

#### 3.5.4. Tablet Friability

The friability of SUL-FDTs was studied and calculated according to the USP 38 guidelines as previously reported and the percentage friability was calculated according to this equation (Equation (8)).

(8) $$\%\;friability=\frac{W1-W2}{W1}\times 100$$

in which *W*1 and *W*2 were the weights of tablets before and after rotation in a friabilator.

#### 3.5.5. In Vitro Disintegration

The disintegration time performance was performed using USP tablet disintegration tester apparatus (Hanson, Variel Ave, Chatsworth, USA). Briefly, tablets were placed in 500 mL distilled water maintained at 37 °C with agitation rate of 30 shakes per min. The tablet was considered disintegrated completely when all the particles passed through the screen. The disintegration time of six individual tablets were recorded and the average was reported for each formulation.

#### 3.5.6. In Vitro Release Study

In vitro release of SUL from the prepared FDTs tablets were performed using an Erweka DT600 dissolution tester type II (paddle method, 100 rpm, Vankel 7000, New Jersey, USA). Dissolution study was performed using phosphate buffer of pH 6.8, 500 mL, at 37 ± 0.5 °C. At different time intervals samples of 5 mL withdrawn after 2, 4, 5, 10, 15, 30, and 45 min and immediately replaced with fresh medium with the same temperature and volume. Samples were then filtered with 0.2 µm membrane filter and SUL concentrations were analysed using UV-VIS spectrophotometry.

### 3.6. In Vivo Study for Optimized Formulation

#### 3.6.1. Study Design and Animals Treatment

In vivo study was performed according to the ethical guidelines stated and approved by the Committee of Medical Ethics (Approval no. S13-20), Faculty of Pharmacy, Assiut University, Egypt. Adult healthy male New Zealand rabbits were divided into three groups (*n* = 5, 1.5 ± 0.2 Kg average rabbit weight). Rabbits were kept at ambient conditions of laboratory temperature (25.0 ± 2.0 °C) and fed in standard diet. The 50 mg SUL human dose was adapted for consideration to determine the equivalent rabbit dose using a specific mathematical equation (Equation (9)) based on the surface area ratio of rabbit to human [35]. The first group received SUL-FDTs containing SUL (4.0 mg). An equivalent weight of SUL dose of 4.0 mg was taken from the commercial SUL capsules and then given to the second group. The third group was used as a negative control. At different time points, samples were collected (1–1.5 mL) from rabbit’s vein located in the marginal ear and placed in a five mL screw-capped centrifuge tubes containing anti-coagulant heparin for 24 h. Further, to separate blood plasma, samples were centrifuged (5000 rpm, 15 min) followed by removal of supernatant and the obtained plasma were deep frozen at −20 °C for further investigation:

(9) $$\mathrm{Human}\;\mathrm{eq}.\;\mathrm{dose}\;(\mathrm{mg}/\mathrm{kg})=\mathrm{Animal}\;\mathrm{dose}\;\left(\mathrm{mg}/\mathrm{kg}\right)\times \frac{\mathrm{Animal}\;\mathrm{Km}}{\mathrm{HUman}\;\mathrm{Km}}$$

where, Km is the ratio of body weight to surface area. Km values for rabbits (0.15 kg) and adult human (60 kg) are 12 and 37, respectively [10,35,36,37].

#### 3.6.2. Samples Preparation for Analysis

Approximately one mL of each plasma sample was mixed with 1–2 mL of acetonitrile and kept for 5–7 min at room temperature. Tubes were gently vortexed, samples were centrifuged at 3500 rpm for fifteen min and SUL concentration was determined from an appropriate amounts collected from the centrifuged supernatant. SUL was determined using the reported method for its determination in blood plasma based on formation of π-π* charge transfer complex between benzene ring of SUL as a rich group of electrons and σ acceptor molecule like the iodine [38]. The produced complex was measured spectrophotometrically at λ_max_ of 360 nm using a computerized UV-Visible spectrophotometer (UV-1601 PC, Shimadzu, Japan) with 1.0 cm quartz cells [38]. The SUL stock solution, standard samples and calibration curve were performed as previously reported [8].

#### 3.6.3. Pharmacokinetic Analysis

The obtained SUL plasma concentration was plotted against the respective time points to obtain the plasma concentration time profile curve. In addition, pharmacokinetic parameters were investigated as previously reported [32,37]. From the obtained plasma concentration time curve both C_max_ and T_max_ were determined. Both the absorption rate constant (K_abs_) and the elimination rate constant (K_el_.) were calculated from the method of residual and the slope of the terminal linear portion of the curve, respectively. Furthermore, dividing 0.693 by the respective rate constant, the apparent half-lives of absorption and elimination (t_½_) were obtained. In addition, (AUC_0–t_) and (AUMC_0–t_) from 0 to 24 h were calculated via the trapezoidal rule. Moreover, (AUC_0–∞_ and AUMC_0–∞_) from time zero to infinity were calculated using (Equations (10) and (11)), respectively:

(10) $${\mathrm{AUC}}_{\left(0–\infty \right)}={\mathrm{AUC}}_{\left(0–t\right)}+\frac{Ct}{Kel}$$

(11) $${\mathrm{AUMC}}_{\left(0–\infty \right)}={\mathrm{AUMC}}_{\left(0–t\right)}+\frac{t*Ct}{Kel}+\frac{Ct}{{Kel}^{2}\;}\;$$

where, *Ct* is the SUL final concentration detected at the end time point (*t*), *K_el_* is the SUL elimination rate constant. AUC is the area under plasma concentration time profile and AUMC is the area under first moment curve. In addition, SUL mean residence time (MRT) was calculated using the following equation (Equation (12)):

(12) $$\mathrm{MRT}=\frac{\mathrm{AUMC}\left(0–\infty \right)}{\mathrm{AUC}\left(0–\infty \right)}$$

Finally, the relative bioavailability (FR%) was obtained via dividing the AUC from time zero to infinity of both SUL-FDTs and the Dogmatil^®^ commercial product as previously calculated [8,39]. Relative bioavailability compares the bioavailability of SUL-FDTs to standard SUL commercial product and expressed as a percentage (Equation (13)).

(13) $$F_{R}(\%)=\frac{\mathrm{AUC}\left(0–\infty \right)\;\mathrm{for}\;\mathrm{SUL}–\mathrm{FDT}}{\mathrm{AUC}\left(0–\infty \right)\;\mathrm{for}\;\mathrm{Dogmatil}}\times 100$$

### 3.7. Statistical Analysis

Statistical analysis was performed regarding the in vivo study to demonstrate the significant difference values for the obtained pharmacokinetic parameters. Comparison between data was investigated using the Student’s *t*-test and the level of significance was recorded at *p* values ≤ 0.05 and *p* ≤ 0.001.

## 4. Conclusions

In this study, FDTs were developed to enhance the oral bioavailability of SUL and to achieve a rapid onset of action by avoiding the P-gp located in different parts of GIT responsible for drug efflux into the intestinal lumen. A full factorial experimental design was successfully utilized to formulate and optimize SUL-FDTs. The optimized SUL-FDTs formulation showed acceptable mechanical strength (4.58 ± 0.52 KP), rapid in vitro disintegration time (37.5 ± 1.87 s) and high release rate (100.51 ± 1.34% after 30 min). According to the in vivo study, the optimized SUL-FDTs ameliorate the poor oral bioavailability of SUL, as demonstrated via significant (*p* < 0.01) increase in C_max_ and AUC_(0–∞)_ compared to the marketed product (Dogmatil^®^ capsules). In addition, the optimized SUL formulation showed a 9.3-fold enhancement in oral SUL bioavailability with a relative bioavailability value of 931.6 ± 36.5%. Thus, the SUL-FDTs formulation could be considered as an alternative to the marketed SUL product with enhanced bioavailability and faster action which could be also useful for patients having problems in swallowing as well as elderly people.

## Figures and Tables

**Table 1:** Critical quality attributes of sulpiride FDTs formulations (Mean ± SD).

Formula	Weight (mg ± SD)	Content Uniformity (% ± SD) (AV)	Breaking Force (KP ± SD)	Friability (% ± SD)	DT (s ± SD)	% Release after 30 min (min ± SD)
**1**	195 ± 3.20	99.8 ± 1.27 (**3.048**)	3.25 ± 0.25	0.84 ± 0.2	15.16 ± 1.47	84.79 ± 2.05
**2**	200.17 ± 1.79	100.3 ± 1.74 (**4.176**)	4.0 ± 0.5	1.01 ± 0.43	33.83 ± 5.98	96.39 ± 2.03
**3**	201.8 ± 1.60	99.33 ± 1.98 (**4.752**)	4.16 ± 0.53	0.53 ± 0.07	21.16 ± 6.85	98.55 ± 1.46
**4**	197.6 ± 149	99.53 ± 1.12 (**4.688**)	4.43 ± 0.11	0.98 ± 0.24	31.66 ± 3.82	87.56 ± 0.45
**5**	199 ± 2.09	97.66 ± 1.45 (**4.32**)	4.9 ± 0.17	0.83 ± 0.3	39.83 ± 2.63	96.58 ± 0.77
**6**	198.8 ± 1.66	98.3 ± 1.9 (**4.76**)	4.9 ± 0.17	0.37 ± 0.15	66.33 ± 5.24	99.39 ± 0.96
**7**	199 ± 2.28	99.03 ± 2.17 (**5.208**)	3.58 ± 0.14	0.58 ± 0.07	40.16 ± 4.35	97.35 ± 0.68
**8**	200 ± 1.26	99.1 ± 3.14 (**7.536**)	3.9 ± 0.17	0.9 ± 0.27	51 ± 6.69	87.47 ± 0.88
**9**	200 ± 2.23	98 ± 2.3 (**6.02**)	3.56 ± 0.11	0.9 ± 0.29	44 ± 14.31	94.15 ± 1.16

**AV:** Acceptance Value and **DT**: Disintegration Time.

**Table 2:** Analysis of variance (ANOVA) summary and regression analysis of SUL-FDTs dependent responses.

**Variables**	**Coefficient Estimate**	**Sum of Squares**	**Standard Error**	***F*** **-Value**	***p*** **-Value**	**95% CI Low**	**95% CI High**
**Hardness (*Quadratic model*)**							
**Model**	-	-	-	496.33	**0.0001**	-	-
**Intercept**	4.93	-	0.0247	-	-	4.86	5.01
**X_1_**	−0.0617	0.0228	0.0135	20.83	**0.0197**	−0.1047	−0.0187
**X_2_**	0.2267	0.30830	0.0135	281.43	**0.0005**	0.1837	0.2697
**X_1_ X_2_**	−0.2325	0.2612	0.0165	197.40	**0.0008**	−0.2852	−0.1798
**Friability (*2FI model*)**							
**Model**	-	-	-	1.180	**0.4054**	-	-
**Intercept**	0.7849	-	0.0739	-	-	0.4959	0.9749
**X_1_**	0.0137	0.0011	0.0905	0.0228	**0.8859**	−0.2190	0.2463
**X_2_**	−0.0900	0.0486	0.0905	0.9887	**0.3657**	−0.3227	0.1427
**X_1_ X_2_**	0.1762	0.1243	0.1109	2.53	**0.1727**	−0.1087	0.4612
**Disintegration time (*linear model*)**							
**Model**	-	-	-	9.98	**0.0124**	-	-
**Intercept**	40.35	-	3.25	-	-	32.39	48.30
**X_1_**	14.17	1204.45	3.98	12.66	**0.0120**	4.43	23.91
**X_2_**	10.75	693.53	3.98	7.29	**0.0356**	1.01	20.49
**Percent release after 30 min (*Quadratic model*)**							
**Model**	-	-	-	68.12	**0.0027**	-	-
**Intercept**	96.31	-	0.5623	-	-	94.52	98.10
**X_1_**	−0.6183	2.29	0.3080	4.03	**0.1383**	−1.6	0.3617
**X_2_**	5.40	175.18	0.3080	307.84	**0.0004**	4.42	6.38
**X_1_ X_2_**	−0.2075	0.1722	0.3772	0.3027	**0.6205**	−1.41	99.28

X_1_ and X_2_ represent the concentration of superdisintegrant and Prosolv^®^ respectively, X_1_X_2_ is the interaction effect.

**Table 3:** The constraints adopted for optimization of tested variables and estimation of overall desirability.

Variables	Target	Range	Weight	Importance Co-Efficient
**In-put**				
Superdisintegrant conc.	In range	5–15%	1	-
Prosolv^®^ conc.	In range	10–40%	1	-
**Out-put**				
Hardness Friability	Maximize 0.7%	3.25–4.9 KP 0.378–1.01%	1	++++ ++++
Disintegration time	30 s	15.16–66.33 s	1	++++
%Release after 30 min	In range	87.47–99.39%	1	-
**Overall desirability = 0.905**				

++++ means “Strength of importance”.

**Table 4:** Predicted and actual values for all dependent responses of optimized formulation with their relative errors.

Responses	Predicted Values	Observed Values (mean ± SD)	Relative Error (%)
**Weight uniformity (mg)**	-	200.51 ± 2.72	-
**Content uniformity (%) (AV)**	-	100.31 ± 1.73 (4.36)	-
**Hardness (KP)**	4.765	4.583 ± 0.52	3.819
**Friability (%)**	0.70	0.731 ± 0.158	−4.428
**Disintegration time (s)**	37.04	37.5 ± 1.87	−1.241
**%Release after 30 min**	98.201	100.514 ± 1.339	−2.355

**Table 5:** Plasma pharmacokinetic parameters of optimized SUL-FDT and SUL commercial product (Dogmatil ^®^). Results expressed as mean ± SD (*n* = 5).

Pharmacokinetic Parameters	Optimized SUL FDT	SUL Commercial Product (Dogmatil^®^)	*p-*Value
C_max1_ (ng/mL)	3299.6 ± 498.8	564.5 ± 25.97	** <0.01
T_max1_ (h)	1.0	0.5	
C_max2_ (ng/mL)	7353.5 ± 471.3	526.2 ± 12.5	** <0.01
T_max2_ (h)	4.0	2.0	* <0.05
C_max3_ (ng/mL)	6561.6 ± 247.2	673.4 ± 51.5	** <0.01
T_max3_ (h)	24.0	24.0	>0.05
K_abs.1_ (h^−1^)	1.85 ± 0.06	3.02 ± 0.16	* <0.05
t_1/2 abs.1_ (h)	0.37 ± 0.09	0.23 ± 0.17	>0.05
K_abs.2_ (h^−1^)	0.35 ± 0.08	0.66 ± 0.16	* <0.05
t_1/2 abs.2_ (h)	2.02 ± 0.12	1.05 ± 0.31	* <0.05
K_abs.3_ (h^−1^)	0.058 ± 0.01	0.056 ± 0.011	>0.05
t_1/2 abs.3_ (h)	12.01 ± 0.15	12.36 ± 0.21	>0.05
AUC_0–24_ (ng·h/mL)	129,948.6 ± 1230	9311 ± 482	** <0.01
AUC_0–∞_ (ng·h/mL)	148,283.8 ± 988.5	15,917.45 ± 675	** <0.01
AUMC_0–24_ (ng·h^2^/mL)	1,745,461.2 ± 2380	193,150.5 ± 1653	** <0.01
AUMC_0–∞_ (ng·h^2^/mL)	2,236,763.4 ± 3458	416,517.6 ± 1816	** <0.01
MRT (h)	15.08 ± 0.78	26.17 ± 0.59	* <0.05
K_el_ (h^−1^)	0.357 ± 0.081	0.318 ± 0.011	>0.05
t_1/2el_ (h) Relative bioavailability (%)	1.93 ± 0.052 931.6 ± 36.5	2.17 ± 0.33 -	>0.05 -

K_el_, t_1/2el_, were calculated from the second phase of drug absorption, which begin after T_max2_ of 4 and 2 h for SUL-FDT and Dogmatil^®^, respectively. * *p* ≤ 0.05 and ** *p* ≤ 0.001.

**Table 6:** Independent formulation variables and their levels used in experimental design.

Coded Levels	Superdisintegrant Conc. (%)	Prosolv^®^ Conc. (%)
−1	5	10
0	10	25
1	15	40

−1: factor at low level; 0: factor at medium level; 1: factor at high level.

**Table 7:** A full matrix of 3^2^ full factorial design for Sulpiride FDT formulations.

Experiment Code	Superdisintegrant Conc. (%)	Prosolv^®^ Conc. (%)
1	5	10
2	5	25
3	5	40
4	10	10
5	10	25
6	10	40
7	15	10
8	15	25
9	15	40

**Table 8:** The quantitative composition of sulpiride FDT formulations.

Ingredients	% *w*/*w*
Sulpiride	25
Croscarmellose sodium (CCS) (Vivasol ^®^)	5, 10 and 15
Silicified microcrystalline cellulose (Prosolve^®^)	10, 25 and 40
Spray dried mannitol	Up to 100
Magnesium stearate	1
